# Hallucinations as a risk marker for suicidal behaviour in individuals with a history of sexual assault: a general population study with instant replication

**DOI:** 10.1017/S0033291722001532

**Published:** 2023-07

**Authors:** Kathryn Yates, Ulla Lång, Evyn M. Peters, Johanna T. W. Wigman, David Boyda, Fiona McNicholas, Mary Cannon, Ben Alderson-Day, Michael Bloomfield, Hugh Ramsay, Ian Kelleher

**Affiliations:** 1 Department of Psychiatry, Royal College of Surgeons in Ireland, Dublin 2, Ireland; 2 Department of Psychiatry, University of Saskatchewan, Saskatoon SK, Canada; 3 Department of Psychiatry, Interdisciplinary Center Psychopathology and Emotion regulation (ICPE), University of Groningen, Groningen, The Netherlands; 4 School of Psychology, University of Wolverhampton, Wolverhampton, UK; 5 School of Medicine, University College Dublin, Belfield, Dublin, Ireland; 6 Lucena Clinic, St John of God Hospitaller Services, Dublin, Ireland; 7 Department of Child Psychiatry, Our Lady's Hospital for Sick Children, Dublin, Ireland; 8 Department of Psychology, Science Laboratories, Durham University, South Road, Durham, UK; 9 Division of Psychiatry, University College London, London, UK; 10 Traumatic Stress Clinic, Camden & Islington NHS Foundation Trust, London, UK; 11 National Institute for Health Research, University College London Hospitals Biomedical Research Centre, London, UK; 12 University College London Hospitals NHS Foundation Trust, London, UK; 13 Department of Psychiatry, Research Unit of Clinical Neuroscience, University of Oulu, Oulu, Finland

**Keywords:** Hallucinations, psychosis, sexual assault, suicidal behaviour, trauma

## Abstract

**Background:**

Research has shown a strong relationship between hallucinations and suicidal behaviour in general population samples. Whether hallucinations also index suicidal behaviour risk in groups at elevated risk of suicidal behaviour, namely in individuals with a sexual assault history, remains to be seen.

**Aims:**

We assessed whether hallucinations were markers of risk for suicidal behaviour among individuals with a sexual assault history.

**Methods:**

Using the cross-sectional 2007 (*N* = 7403) and 2014 (*N* = 7546) Adult Psychiatric Morbidity Surveys, we assessed for an interaction between sexual assault and hallucinations in terms of the odds of suicide attempt, as well as directly comparing the prevalence of suicide attempt in individuals with a sexual assault history with *v.* without hallucinations.

**Results:**

Individuals with a sexual assault history had increased odds of hallucinations and suicide attempt compared to individuals without a sexual assault history in both samples. There was a significant interaction between sexual assault and hallucinations in terms of the odds of suicide attempt. In total, 14–19% of individuals with a sexual assault history who did not report hallucinations had one or more suicide attempt. This increased to 33–52% of individuals with a sexual assault history who did report hallucinations (2007, aOR = 2.85, 1.71–4.75; 2014, aOR = 4.52, 2.78–7.35).

**Conclusions:**

Hallucinations are a risk marker for suicide attempt even among individuals with an elevated risk of suicidal behaviour, specifically individuals with a sexual assault history. This finding highlights the clinical significance of hallucinations with regard to suicidal behaviour risk, even among high-risk populations.

Individuals with a history of sexual assault are at significantly elevated risk of attempted and death by suicide (Dworkin, DeCou, & Fitzpatrick, 2020). The US Surgeon General and others have highlighted the need for further research to identify risk markers for suicidal behaviour specifically among individuals with a history of sexual assault (US Office of the Surgeon General, 2012). However, there has been little progress on this to date.

Research has shown evidence of a robust association between hallucinations and severity of childhood sexual trauma, in excess of the relationship between hallucinations and total childhood trauma or neglect (Bailey et al., 2018). The strength of this relationship has been demonstrated both in childhood and adulthood (Hardy et al., 2005). What is more, there is now also a great deal of research demonstrating a strong relationship between hallucinations and risk of suicidal behaviour (Hielscher et al., 2019; Honings, Drukker, Groen, & van Os, 2016; Kelleher et al., 2013; Toyohara et al., 2021). In a systematic review and meta-analysis of prospective cohort studies, we found that individuals who reported hallucinations had a 3-fold increased odds of suicide attempt and a 4-fold increased odds of suicide death (Yates et al., 2019). While general population research has repeatedly shown a strong relationship between hallucinations and suicidal behaviour, it remains to be seen whether hallucinations are also a risk marker for suicidal behaviour specifically among high-risk groups – namely, individuals with a history of sexual assault. Some researchers have suggested that adverse experiences, such as sexual assault, may be mediators of the relationship between hallucinations and suicidal behaviour (Hielscher et al., 2021; Narita, Wilcox, & DeVylder, 2020). This suggests that the relationship between hallucinations and suicidal behaviour would be eliminated or substantially attenuated once sexual assault is accounted for.

We hypothesised, on the other hand, that even when limiting analyses on the relationship between hallucinations and suicidal behaviour solely to individuals with a history of sexual assault, hallucinations would still act as a strong marker of risk for suicidal behaviour. That is, comparing individuals who have a history of sexual assault who also report hallucinations to individuals who also have a history of sexual assault but who, in contrast, do not report hallucinations. We tested this hypothesis using data on hallucinations, sexual assault and suicidal behaviour in the 2007 UK Adult Psychiatric Morbidity Survey (APMS). We tested the reliability of our findings by carrying out instant replication of our analyses using equivalent data from the 2014 APMS study.

## Methods

The APMS are national household surveys that assess the cross-sectional prevalence of treated and untreated mental health disorders in an adult sample representative of the general UK population. The surveys have been conducted every seven years since 1993 (2000, 2007, 2014) using consistent methods. At each of the four APMS assessment time points, approximately 7500 people aged 16 years and over were interviewed after having been identified using household probability sampling. The first two surveys, conducted in 1993 and 2000, collected data on individuals aged 16–74 from England, Scotland and Wales and were conducted by the Office of National Statistics. The 2007 and 2014 surveys collected data from individuals aged 16 years onwards with no upper age limit in England only and were conducted by NatCen Social Research, in collaboration with the University of Leicester.

The survey uses a two-phase approach. During the first phase, trained interviewers collect data on demographic, social and service use variables, as well as a number of physical and mental health disorders. The second phase, conducted by clinically trained interviewers, collects data on suicidality and self-harm behaviours as well as adverse and psychotic experiences.

In line with previous studies using the APMS datasets, all analyses were weighted to ensure the data was representative of the general population (Marwaha, Broome, Bebbington, Kuipers, & Freeman, 2014; McManus, Meltzer, Brugha, Bebbington, & Jenkins, 2009). For full APMS methods see McManus, Bebbington, Jenkins, and Brugha (2016). After running the analyses in the 2007 sample, identical analyses were ran on the 2014 sample to conduct an instant replication.

### Measures

#### Lifetime sexual assault

The following standalone questions were used to assess sexual assault in the APMS 2007 and the APMS 2014:

Before the age of 16…

‘…*did anyone touch you, or get you to touch them, in sexual way without your consent*?’

*‘… has anyone had sexual intercourse with you without your consent’.*

Since the age of 16…

‘…*did anyone touch you, or get you to touch them, in sexual way without your consent*?’

*‘… has anyone had sexual intercourse with you without your consent’.*

#### Hallucinations

Hallucinations were assessed using the following question from the Psychosis Screening Questionnaire (Bebbington & Nayani, 1995; Chaumette et al., 2016): ‘*Over the past year, have there been times when you heard or saw things that other people couldn't?’* Research has shown questionnaire items on auditory and visual hallucinations have high positive and negative predictive value for interview verified symptoms (Gundersen et al., 2019; Kelleher, Harley, Murtagh, & Cannon, 2011).

Individuals who endorsed at least one of the below screening criteria were invited for an assessment using the Schedules for Clinical Assessment in Neuropsychiatry (SCAN), as their answers indicated a possible psychotic disorder (Wing et al., 1990): current antipsychotic use, history of psychiatric hospital admission, self-reported diagnosis or symptoms suggesting a psychotic disorder, or a positive response to the question ‘Did you at any time hear voices saying quite a few words or sentences when there was no one around that might account for it?’. Individuals who met SCAN criteria for a psychotic disorder or individuals who were unable to be assessed but endorsed at least two of the above screening criteria were categorised as having a probable psychotic disorder. As a more stringent test of our hypothesis, individuals with a probable psychotic disorder were excluded from the final model (See Statistical Analysis section for more details).

### Outcome measures

#### Suicidality

Questions assessing lifetime suicidal ideation and attempt respectively were as follows: ‘Have you ever thought of taking your life, even if you would not really do it?’ and ‘Have you ever made an attempt to take your life, by taking an overdose of tablets or in some other way?’ (McManus et al., 2009, 2016).

### Covariates

#### Adjusting for other forms of psychopathology

We investigated whether hallucinations were a potential risk marker for suicidal behaviour beyond common measures of psychopathology. Past week prevalence of the following psychiatric symptoms were assessed using the Clinical Interval Schedule (CIS-R; Brugha et al., 1999): depression, phobia, panic disorder, obsessive compulsive disorder (OCD), mixed anxiety and depression, and generalised anxiety disorder. Total CIS-R psychopathology score was added to the model to examine for risk marker effects of hallucinations in excess of those indexed by co-occurring general psychopathology.

Given that hallucinatory experiences and suicidal behaviour are both associated with borderline personality disorder (BPD) traits, as an additional stringent test of our hypothesis we also added scores for borderline personality traits to our final model, using the screening version of the Structured Clinical Interview for DSM-IV Axis II Personality Disorders (SCID-II; First, Gibbon, Spitzer, Williams, & Benjamin, 1997). The SCID-II borderline section contains 15 items. Two of these items were removed as they specifically measure suicidal behaviour. The remaining 13 items were summed to create a dimensional borderline personality score.

### Statistical analysis

We used logistic regression to examine the prevalence of suicidal ideation and suicide attempt in individuals with *v.* without exposure to sexual assault.

In order to test whether hallucinations were a risk marker for suicidal behaviour, in our main analyses we compared the prevalence of suicide attempt among individuals who reported sexual assault who did report and who did not report past-year hallucinations. We used logistic regressions to examine the odds of suicide attempt in individuals who reported both sexual assault and past-year hallucinations compared to individuals who reported sexual assault but who did not report past-year hallucinations. We conducted interaction analyses using the Wald test to see whether there was an interaction between sexual assault and hallucinations on the odds of suicide attempt.

All analyses were adjusted for age and sex and conducted using StataSE 14. Analyses in text are adjusted for age and sex. In the final model, we additionally adjusted for CIS-R and BPD score. In the supplement, we ran analyses excluding individuals with probable psychotic disorder (2007 *N* = 40, 0.54% of total sample; 2014 *N* = 91, 1.21% of total sample) to ensure that any significant relationships found were not due to psychotic disorder.

The authors assert that all procedures contributing to this work comply with the ethical standards of the relevant national and institutional committees on human experimentation and with the Helsinki Declaration of 1975, as revised in 2008. All procedures involving human subjects/patients were approved by The Royal Free Medical School Research Ethics Committee (2007 survey, Ref.: 06/Q0501/71) and the West London REC (2014 survey, Ref.: 14/LO/0411).

## Results

### General descriptive statistics

The total sample for the 2007 dataset was *n* = 7403. The age range of respondents was 16 to 95 years (*M* = 51.1, s.d. = 18.6). The majority of the respondents were female (56.8%, *n* = 4206). The prevalence of past year hallucinations was 4.37% (*n* = 323). Approximately 13% of the sample (*n* = 934) reported sexual assault at any age.

The total sample for the 2014 dataset was *n* = 7546. The age range of respondents was 16 to 95 years (*M* = 52.3, s.d. = 18.8). The majority of the respondents were female (59.5%, *n* = 4488). The prevalence of past year hallucinations was 4.59% (*n* = 346). Approximately 13% of the sample (*n* = 917) reported sexual assault at any age.

### Prevalence of hallucinations, suicidal ideation and suicide attempt by sexual assault

#### 2007 dataset

Eight per cent of individuals with a sexual assault history reported past year hallucinations, compared with 4% of individuals without a sexual assault history (aOR = 2.11, 95%CI = 1.61–2.78). Thirty six per cent of individuals with a sexual assault history reported lifetime suicidal ideation, compared with 11% of individuals without a sexual assault history (aOR = 4.00, 95%CI = 3.41–4.69). Sixteen per cent of individuals with a sexual assault history reported one or more lifetime suicide attempts, compared with 4% of individuals without a sexual assault history (aOR = 4.37, 95%CI = 3.48–5.47).

#### 2014 dataset

Eight per cent of individuals with a sexual assault history reported past year hallucinations, compared with 4% of individuals without a sexual assault history (aOR = 2.16, 95%CI = 1.64–2.85). Forty-seven per cent of individuals with a sexual assault history reported lifetime suicidal ideation, compared with 18% of individuals without sexual assault history (aOR = 3.97, 95%CI = 3.42–4.61). Twenty-two per cent of individuals with a sexual assault history reported at least one lifetime suicide attempt, compared with 5% of individuals without a sexual assault history (aOR = 4.99, 95%CI = 4.08–6.10).

### Main analyses

In both the 2007 and 2014 datasets, individuals with a history of sexual assault who also reported hallucinations were at increased odds of suicide attempt compared to individuals with a history of sexual assault but who did not report hallucinations (See Table 1). There was a significant interaction between sexual assault and hallucinations, such that hallucinations indexed risk for suicide attempt in excess of that explained by sexual assault [*p* < 0.001 in both the 2007 and 2014 dataset]. Whereas, in the 2007 study, 14% of individuals with a history of sexual assault who did not report hallucinations had one or more suicide attempt, 33% of individuals with a history of sexual assault who reported hallucinations had one or more suicide attempt. In the 2014 study, 19% of individuals with a history of sexual assault who did not report hallucinations had one or more suicide attempt compared to 52% of individuals with a history of sexual assault who reported hallucinations.

**Table 1.** Relationship between hallucinations and suicide attempt in individuals with a history of sexual assault

	Sexual assault +ve	Sexual assault +ve	OR^a^ (95%CI)	OR^b^ (95%CI)
	Hallucinations −ve *n*(%)	Hallucinations +ve *n*(%)		
2007	122 (14)	25 (33)	**2.85 (1.71–4.75)**	**1.92 (1.08–3.39)**
2014	162 (19)	39 (52)	**4.52 (2.78–7.35)**	**1.85 (1.06–3.22)**

−ve, negative; +ve, positive; OR, odds ratio; 95%CI, 95% confidence interval. Bold font indicates statistical significance.

a Adjusted for age and sex.

b Adjusted for age, sex, total CIS-R and BPD Score.

Table 2 shows the prevalence of suicidal ideation and suicide attempts across four groups in the 2007 dataset: (1) individuals with neither a history of sexual assault nor hallucinations (reference group), (2) individuals without a history of sexual assault but who did report past-year hallucinations, (3) individuals with a history of sexual assault but who did not report past-year hallucinations, and (4) individuals with a history of sexual assault and who also reported hallucinations.

**Table 2.** Suicidal ideation and attempt stratified by sexual assault history and hallucinations, 2007

	Sexual assault −ve		Sexual assault −ve			Sexual assault +ve			Sexual assault +ve		
	Hallucinations −ve		Hallucinations +ve			Hallucinations −ve			Hallucinations +ve		
	*N* (%)	OR (95%CI)	*N* (%)	OR^a^ (95%CI)	OR^b^ (95%CI)	*N* (%)	OR^a^ (95%CI)	OR^b^ (95%CI)	*N* (%)	OR^a^ (95%CI)	OR^b^ (95%CI)
Suicidal ideation	651 (10.7)	1 (ref)	74 (31)	**3.62** (**2.71–4.83)**	**1.58 (1.12–2.24)**	286 (33.4)	**3.88 (3.28–4.60)**	**2.46 (2.01–3.01)**	49 (65.3)	**14.11 (8.68–22.95)**	**5.89 (3.17–10.94)**
Suicide attempt	203 (3.3)	1 (ref)	29 (12.1)	**3.86** (**2.55–5.82)**	**1.65 (1.03–2.65)**	122 (14.2)	**4.30 (3.37–5.49)**	**2.57 (1.95–3.40)**	25 (33.3)	**12.36 (7.54–20.27)**	**4.47 (2.37–8.41)**

−ve, negative; +ve, positive; SA, sexual assault; OR, odds ratio; 95%CI, 95% confidence interval. Bold font indicates statistical significance.

a Analyses adjusted for age and sex.

b Adjusted for age, sex, CIS-R score and BPD score.

Table 3 shows the prevalence of suicidal ideation and suicide attempts across four groups in the 2014 dataset: (1) individuals with neither a history of sexual assault nor hallucinations (reference group), (2) individuals without a history of sexual assault but who did report past-year hallucinations, (3) individuals with a history of sexual assault but who did not report past-year hallucinations, and (4) individuals with a history of sexual assault and who also reported hallucinations.

**Table 3.** Suicidal ideation and attempt stratified by sexual assault history and hallucinations, 2014

	Sexual assault −ve		Sexual assault −ve			Sexual assault +ve			Sexual assault +ve		
	Hallucinations −ve		Hallucinations +ve			Hallucinations −ve			Hallucinations +ve		
	*N* (%)	OR (95%CI)	*N* (%)	OR^a^ (95%CI)	OR^b^ (95%CI)	*N* (%)	OR^a^ (95%CI)	OR^b^ (95%CI)	*N* (%)	OR^a^ (95%CI)	OR^b^ (95%CI)
Suicidal ideation	987 (17)	1 (ref)	109 (44.9)	**3.87** (**2.97–5.03)**	**1.72 (1.17–2.53)**	379 (45)	**3.97 (3.40–4.64)**	**3.14 (2.55–3.87)**	51 (68)	**9.94 (6.11–16.18)**	**2.05 (0.99–4.23)**
Suicide attempt	269 (4.7)	1 (ref)	43 (17.7)	**4.25** (**2.98–6.06)**	**1.72 (1.10–2.69)**	162 (19.2)	**4.71 (3.79–5.85)**	**3.00 (2.28–3.94)**	39 (52)	**21.04 (13.18–33.58)**	**4.97 (2.82–8.76)**

−ve, negative; +ve, positive; OR, odds ratio; 95%CI, 95% confidence interval. Bold font indicates statistical significance.

a Analyses adjusted for age and sex.

b Adjusted for age, sex, CIS-R score and BPD score.

See online Supplementary Tables S1–S3 for this analysis excluding individuals with probable psychotic disorder. In the 2007 sample, individuals with a history of sexual assault who also reported hallucinations were at increased odds of suicide attempt compared to individuals with a history of sexual assault but who did not report hallucinations. Relationships remained significant when adjusting for total CIS-R and BPD score. In the 2014 sample, individuals with a history of sexual assault who also reported hallucinations were at increased odds of suicide attempt compared to individuals with a history of sexual assault but who did not report hallucinations. However, when adjusting for total CIS-R and BPD score the lower confidence interval went below one.

## Discussion

Individuals with a history of sexual assault are at elevated risk of attempted and death by suicide (Angelakis, Gillespie, & Panagioti, 2019; Dworkin et al., 2020; Zatti et al., 2017) but there is a lack of data on risk markers that might identify particularly vulnerable individuals amongst this cohort. In line with previous research, we found that individuals with a history of sexual assault had significantly increased odds of attempted suicide. Individuals with a history of sexual assault also had a significantly increased odds of hallucinations. There was a significant interaction between sexual assault and hallucinations, such that hallucinations indexed risk for suicide attempt in excess of that explained by sexual assault. Whereas 14% of individuals with a history of sexual assault who did not report hallucinations had one or more suicide attempt, 33% of individuals with a history of sexual assault who reported hallucinations had one or more suicide attempt (2007 study). In the 2014 study, 19% of individuals with a history of sexual assault who did not report hallucinations had one or more suicide attempt whereas more than half (52%) of individuals with a history of sexual assault with hallucinations had one or more suicide attempt. Hallucinations, therefore, represent a strong risk marker for suicide attempt in individuals with a history of sexual assault.

Excluding individuals with probable psychotic disorder from our analyses made little difference to the results and overall there were no differences compared to the main analyses.: among individuals with both a history of sexual assault and who reported hallucinations, 30% had one or more suicide attempt in the 2007 dataset and 43% had one or more suicide attempt in the 2014 dataset. This shows that the results are not driven by psychotic disorder but, rather, that even ‘subclinical’ hallucinatory experiences might act as a risk marker for suicidal behaviour in individuals who have a history of sexual assault.

There are a number of possible mechanisms underlying the strong relationship between hallucinations and suicidal behaviour found in this study. From a biological perspective, trauma is associated with differences in a number of brain areas and functions, one of which is brain connectivity (Teicher, Samson, Anderson, & Ohashi, 2016). For example, exposure to sexual abuse in childhood is associated with functional differences in the limbic system and, in one study, has been shown to predict amygdala hyper-reactivity to negative mood induction [this was done by asking participants to listen to music associated with sad mood whilst reading a sad autobiographical script they had written previously (Cassiers et al., 2018; Yamamoto et al., 2017)]. Importantly, these brain areas underpin cognitive functions such as executive and emotional processing, which are associated with both hallucinations and suicide risk.

From a psychosocial perspective, cognitive biases as a result of trauma may be related to psychosis risk (Gibson, Alloy, & Ellman, 2016; Livet, Navarri, Potvin, & Conrod, 2020). These biases are also found in suicidal individuals. For example, suicide-related rumination and attentional bias towards suicide-related stimuli have been associated with increased risk for suicidal behaviour (Cha, Najmi, Park, Finn, & Nock, 2010; Rogers & Joiner, 2018). Similarly, in a sample of patients with a first episode of psychosis, Cui et al. (2019) found that negative schema significantly mediated the relationship between childhood trauma and suicidality. It could be that hallucinations are marking the development of negative schemas and other cognitive biases, which increase risk for suicidal behaviour. Feelings of shame have also been associated with hallucinations and recent experimental research has shown that hallucinations themselves elicit shame in a general population-based sample (Bortolon et al., 2022; McCarthy-Jones, 2017). A recent meta-analysis has shown that feelings of shame are associated with an increased risk of suicidal behaviour (Sheehy et al., 2019).

Exposure to sexual assault may also negatively affect the development of emotional regulation skills which may be associated with both hallucinations and suicidal behaviour (Bloomfield et al., 2021; Kim & Cicchetti, 2010). However, we adjusted our analyses for emotional dysregulation and other borderline personality traits and found that this did not explain the relationship between hallucinations and suicidal behaviour. However, it is important to note that we do not view these phenomena as confounders in the relationship between hallucinations and suicidal behaviour. Evidence supports a mediating role of mental disorders in these relationships (Hielscher, DeVylder, Saha, Connell, & Scott, 2018; Jang et al., 2014; Núñez, Monjes, Campos, & Wigman, 2021). We wished to investigate whether emotional dysregulation and other borderline personality traits fully mediated (i.e. explained) or partially mediated the relationship between hallucinations and suicide attempt. Deeper exploration of their role in this association is out of scope for this cross-sectional paper.

Kelleher and Cannon (2021) recently suggested that hallucinations may represent ‘a measurable marker of the subjective impact of adverse life events’; that is, that hallucinations may offer insight into the subjective traumatic impact or severity of moral injury of adverse experiences, such as sexual assault. Our finding that hallucinations were strong risk markers for attempted suicide among individuals with a history of sexual assault is in keeping with this theory.

It is important to note that individuals with a history of sexual assault have a baseline high risk for suicidal behaviour (Monteith, Holliday, Schneider, Forster, & Bahraini, 2019). However, additional knowledge about hallucinations increases knowledge about risk to a degree that is likely to impact on risk mitigation strategies (e.g. urgency of review and treatment). We found that the prevalence of suicide attempt in individuals with a history of sexual assault escalated from 14–19% in individuals without hallucinations to 33–52% in individuals with hallucinations. This information is particularly valuable in clinical settings providing support for individuals with a history of sexual assault.

It is further clinically relevant that the association remained significant after controlling for psychopathology and borderline personality scores. This finding indicates that the association between hallucinations and suicidal behaviour in individuals with a history of sexual assault is not simply detecting risk arising from general psychopathology or borderline personality traits.

## Strengths and limitations

A key strength of the current study was that we instantly replicated our analyses in an independent sample. The consistency in results across both samples gives us added confidence in the robustness of our findings. Because the data were cross sectional, we cannot determine the direction of the relationship between sexual assault and hallucinations. In addition, suicide variables were lifetime measures, making it difficult to establish temporal relations. Future studies should consider the timeframe of each measure. However, our analyses made no assumptions about causality – rather, we tested whether hallucinations were a risk marker for suicidal behaviour in individuals with a history of sexual assault. The direction of the relationship between hallucinations and sexual assault does not detract from the clinical utility of recognising hallucinations as a risk marker for suicidal behaviour in individuals with a history of sexual assault. Future cross-cultural replication studies are warranted, for example in non-English samples. We did not have information on the frequency of exposure to sexual assault. Future research would be valuable in order to understand these relationships with respect to the transition from suicidal ideation to attempts. Future research focusing on dose-response relationships would also be valuable, as chronic exposure to sexual assault may have a differential impact on suicide outcomes.

Future longitudinal research will be also beneficial, including research on whether trauma-focused treatments reduce both hallucinations and suicidal behaviour risk. In addition, further qualitative research on whether subtypes or subcharacteristics of hallucinations are particularly predictive of attempted suicide will also be valuable. Future research might explore socio-demographic and socioeconomic covariates and also look at a broader range of psychotic experiences and how these associate with suicidal behaviour in the context of sexual assault.

## Conclusion

In two large, representative, general population samples, we found that, hallucinations were a strong marker of risk for suicide attempt in individuals with a history of sexual assault - a group known to be at increased risk of suicidal behaviour. Our findings suggest hallucinations may represent a valuable clinical marker for identifying individuals at increased risk of suicidal behaviour even among groups with an elevated baseline risk, such as individuals with a history of sexual assault.

## Data Availability

The 2007 data that support the findings of this study are openly available in The UK Data Service at http://doi.org/10.5255/UKDA-SN-6379-2. The 2014 data that support the findings of this study are available from NHS Digital. Restrictions apply to the availability of these data, which were used under licence for this study. Data are available The UK Data Service at http://doi.org/10.5255/UKDA-SN-8203-2 with the permission of NHS Digital.

## Supplementary material

For supplementary material accompanying this paper visit https://doi.org/10.1017/S0033291722001532. click here to view supplementary material
