# Supplementary material for: Hallucinations as a risk marker for suicidal behaviour in individuals with a history of sexual assault: a general population study with instant replication
Source: Psychol Med. 2022 Jun 14;53(10):4627–33. doi: 10.1017/S0033291722001532 (PMC10388314; doi:10.1017/S0033291722001532)
Supplement: Supplementary file 1 [file S0033291722001532sup001.docx]

**Hallucinations as risk markers for suicidal behaviour in individuals with a history of sexual assault: a general population study with instant replication**

**Results – excluding individuals with probable psychotic disorder**

***General descriptive statistics – excluding individuals with* probable psychotic disorder**

The total sample for the 2007 dataset was N = 7,403. Individuals with probable psychotic disorder (n = 40) were excluded before analysis (0.54% of the total sample), leaving a total of N = 7,363 for analyses. The age range of respondents was 16-95 (M =51.2, SD =18.61). The majority of the respondents were female (56.8%, n = 4,179). The prevalence of past year hallucinations was 4.13% (n = 304). Approximately 12.7% of the sample (n=920) reported sexual assault.

The total sample for the 2014 dataset was N = 7,546. Individuals with probable psychotic disorder (n = 91) were excluded before analysis (1.21% of the total sample), leaving a total of N = 7,455 for analyses. The age range of respondents was 16-95 (M = 52.4, SD = 18.83). The majority of the respondents were female (59.5%, n = 4,434). The prevalence of past year hallucinations was 4.15% (n = 309). Approximately 12.8% of the sample (n=883) reported sexual assault.

***Prevalence of hallucinations, suicidal ideation and suicide attempt by sexual assault, excluding individuals with probable psychotic disorder***

*2007 Dataset*

Seven percent of individuals with a sexual assault history reported past year hallucinations, compared with 4% of individuals without a sexual assault history (OR = 2.01, 95%CI = 1.51-2.68). Thirty five percent of individuals with a sexual assault history reported lifetime suicidal ideation, compared with 11% of individuals without a sexual assault history (OR = 3.95, 95%CI = 3.36-4.64). Fifteen percent of individuals with a sexual assault history reported one or more lifetime suicide attempts, compared with 4% of individuals without a sexual assault history (OR = 4.26, 95%CI = 3.37-5.37).

*2014 dataset*

Seven percent of individuals with a sexual assault history reported past year hallucinations, compared with 4% of individuals without a sexual assault history (OR = 1.99, 95%CI = 1.48-2.68). Forty-five percent of individuals with a sexual assault history reported lifetime suicidal ideation, compared with 18% of individuals without sexual assault history (OR = 3.76, 95%CI = 3.23-4.38). Twenty percent of individuals with a sexual assault history reported at least one lifetime suicide attempt, compared with 5% of individuals without a sexual assault history (OR = 4.53, 95%CI = 3.68-5.59).

***Main analyses***

In the 2007 dataset, individuals with a history of sexual assault who also reported hallucinations were at increased odds of suicide attempt compared to individuals with a history of sexual assault but who did not report hallucinations. In the 2014 dataset, individuals with a history of sexual assault who also reported hallucinations were at increased odds of suicide attempt compared to individuals with a history of sexual assault but who did not report hallucinations. However, when adjusting for total CIS-R and BPD score the lower confidence interval went below one (see Table S1).

In the 2007 study, 14% individuals with a history of sexual assault who did not report hallucinations disclosed a suicide attempt. This increased to 30% of individuals with a history of sexual assault who did report hallucinations (aOR=2.55, 95%CI=1.47-4.41). Similarly, in the 2014 study, 18% of individuals with a history of sexual assault who did not report hallucinations disclosed a suicide attempt. This increased to 43% of individuals with a history of sexual assault who did report hallucinations (aOR=3.46, 95%CI=2.03-5.88). See below Table S2 and S3 for the prevalence of suicidal ideation and suicide attempt stratified by hallucinations and sexual assault.

**Table S1**. Relationship between hallucinations and suicide attempt in individuals with a history of sexual assault

|  | **Sexual Assault +ve** | **Sexual Assault +ve** | **OR^1^ (95%CI)** | **OR^2^ (95%CI)** |
| --- | --- | --- | --- | --- |
|  | **Hallucinations –ve**  **n(%)** | **Hallucinations +ve**  **n(%)** |  |  |
| 2007 | 116 (14) | 20 (30) | **2.55**  **(1.47-4.41)** | **1.93**  **(1.05-3.52)** |
|  |  |  |  |  |
| 2014 | 146 (18) | 27 (43) | **3.46**  **(2.03-5.88)** | 1.69  (0.93-3.07) |
|  |  |  |  |  |

-ve, negative; +ve, positive; OR, odds ratio; 95%CI, 95% confidence interval.

1. Adjusted for age and sex
2. Adjusted for age, sex, total CIS-R and BPD Score

***Suicidal ideation and attempt in individuals with a sexual assault history, by hallucinations – excluding individuals with probable psychotic disorder***

**Table S2** shows the prevalence of suicidal ideation and suicide attempts across four groups in the 2007 dataset: (1) individuals with neither a history of sexual assault nor hallucinations (reference group), (2) individuals without a history of sexual assault but who did report past-year hallucinations, (3) individuals with a history of sexual assault but who did not report past-year hallucinations, and (4) individuals with a history of sexual assault and who also reported hallucinations.

|  | Sexual assault –ve | | Sexual assault -ve | | | Sexual assault +ve | | | Sexual assault +ve | | |
| --- | --- | --- | --- | --- | --- | --- | --- | --- | --- | --- | --- |
|  | Hallucinations –ve | | Hallucinations +ve | | | Hallucinations -ve | | | Hallucinations +ve | | |
|  | N (%) | OR (95%CI) | N (%) | OR^1^  (95%CI) | OR^2^  (95%CI) | N (%) | OR^1^  (95%CI) | OR^2^  (95%CI) | N (%) | OR^1^  (95%CI) | OR^2^  (95%CI) |
| Suicidal ideation | 643 (10.6) | 1  (ref) | 66 (28.8) | **3.31**  **(2.45-4.48)** | **1.51**  **(1.06-2.17)** | 280 (33) | **3.86**  **(3.25-4.48)** | **2.45**  **(2.00-2.99)** | 42  (62.7) | **12.81**  **(7.72-21.25)** | **6.08**  **(3.24-11.42)** |
| Suicide attempt | 196 (3.2) | 1  (ref) | 25  (11) | **3.57**  **(2.30-5.53)** | **1.62**  **(0.98-2.66)** | 116 (13.6) | **4.23**  **(3.30-5.53)** | **2.54**  **(1.91-3.37)** | 20  (29.9) | **10.97**  **(6.44-18.72)** | **4.63**  **(2.38-9.01)** |

**Table S2:** Suicidal ideation and attempt stratified by sexual assault history and hallucinations, 2007

-ve, negative; +ve, positive; OR, odds ratio; 95%CI, 95% confidence interval.

1. Analyses adjusted for age and sex

2. Adjusted for age, sex , CIS-R score and BPD score

**Table S3.** shows the prevalence of suicidal ideation and suicide attempts across four groups in the 2014 dataset: (1) individuals with neither a history of sexual assault nor hallucinations (reference group), (2) individuals without a history of sexual assault but who did report past-year hallucinations, (3) individuals with a history of sexual assault but who did not report past-year hallucinations, and (4) individuals with a history of sexual assault and who also reported hallucinations.

|  | Sexual assault –ve | | Sexual assault -ve | | | Sexual assault +ve | | | Sexual assault +ve | | |
| --- | --- | --- | --- | --- | --- | --- | --- | --- | --- | --- | --- |
|  | Hallucinations –ve | | Hallucinations +ve | | | Hallucinations -ve | | | Hallucinations +ve | | |
|  | N (%) | OR (95%CI) | N (%) | OR^1^  (95%CI) | OR^2^  (95%CI) | N (%) | OR^1^  (95%CI) | OR^2^  (95%CI) | N (%) | OR^1^  (95%CI) | OR^2^  (95%CI) |
| Suicidal ideation | 974 (16.9) | 1  (ref) | 98 (43.2) | **3.65**  **(2.78-4.80)** | **1.72**  **(1.15-2.57)** | 359 (43.8) | **3.82**  **(3.26-4.48)** | **3.10**  **(2.51-3.82)** | 39 (61.9) | **7.73**  **(4.65-12.87)** | 1.96  (0.92-4.17) |
| Suicide attempt | 262 (4.5) | 1  (ref) | 34  (15) | **3.56**  **(2.41-5.26)** | 1.48  (0.91-2.41) | 146 (17.8) | **4.38**  **(3.50-5.48)** | **2.93**  **(2.22-3.86)** | 27  (42.9) | **14.96**  **(9.00-24.88)** | **4.30**  **(2.30-8.01)** |

**Table S3:** Suicidal ideation and attempt stratified by sexual assault history and hallucinations, 2014

--ve, negative; +ve, positive; OR, odds ratio; 95%CI, 95% confidence interval.

1. Analyses adjusted for age and sex

2. Adjusted for age, sex , CIS-R score and BPD score
